# When Influenza, Bacterial Pneumonia, and COVID-19 Co-exist

**DOI:** 10.7759/cureus.32686

**Published:** 2022-12-19

**Authors:** Modupeoluwa Owolabi, Ruhma Ali, Jenna Dacosta, Ala Muhanna, Jihad Slim

**Affiliations:** 1 Internal Medicine, Saint Michael’s Medical Center, Newark, USA; 2 Infectious Diseases, Saint Michael’s Medical Center, Newark, USA

**Keywords:** co-existing covid-19, case series of influenza, bacterial pneumonia, covid-19, influenza

## Abstract

In the United States, influenza virus and bacterial pneumonia are known to be the leading causes of hospitalization in the winter season. Although healthcare workers are knowledgeable about the management of these co-infections, with the coronavirus disease 2019 (COVID-19) global pandemic that occurred in 2019, a significant change has occurred. The symptoms and clinical manifestations of COVID-19 are similar to that of influenza virus and bacterial pneumonia which can present a unique challenge for healthcare workers. Many reports are available for influenza virus and bacterial pneumonia but none about influenza, bacterial pneumonia, and COVID-19 co-infection.

Here, we present the case of a patient who was admitted with COVID-19, influenza, and bacterial pneumonia co-infection, along with his clinical characteristics, laboratory findings, treatment plan, and outcomes.

## Introduction

In late 2019, people in Wuhan, China started to present with an unknown type of pneumonia, which marked the beginning of the coronavirus disease 2019 (COVID-19) global pandemic. There have been 630,601,291 confirmed cases of COVID-19 worldwide and about 6,583,588 deaths as of November 2022 [1]. The symptoms, mode of transmission, and clinical manifestations of COVID-19 are similar to that of the influenza virus. In contrast, bacterial pneumonia is often transmitted via micro-aspiration. It has been reported that influenza can cause co-infections with other respiratory pathogens [2]; however, there are limited data available on the morbidity and mortality of patients who present with COVID-19, influenza, and bacterial pneumonia co-infection.

In this case report, we present a patient who was admitted with COVID-19, influenza, and bacterial pneumonia co-infection, along with his clinical outcome and treatment plan.

## Case presentation

**Figure 1:**
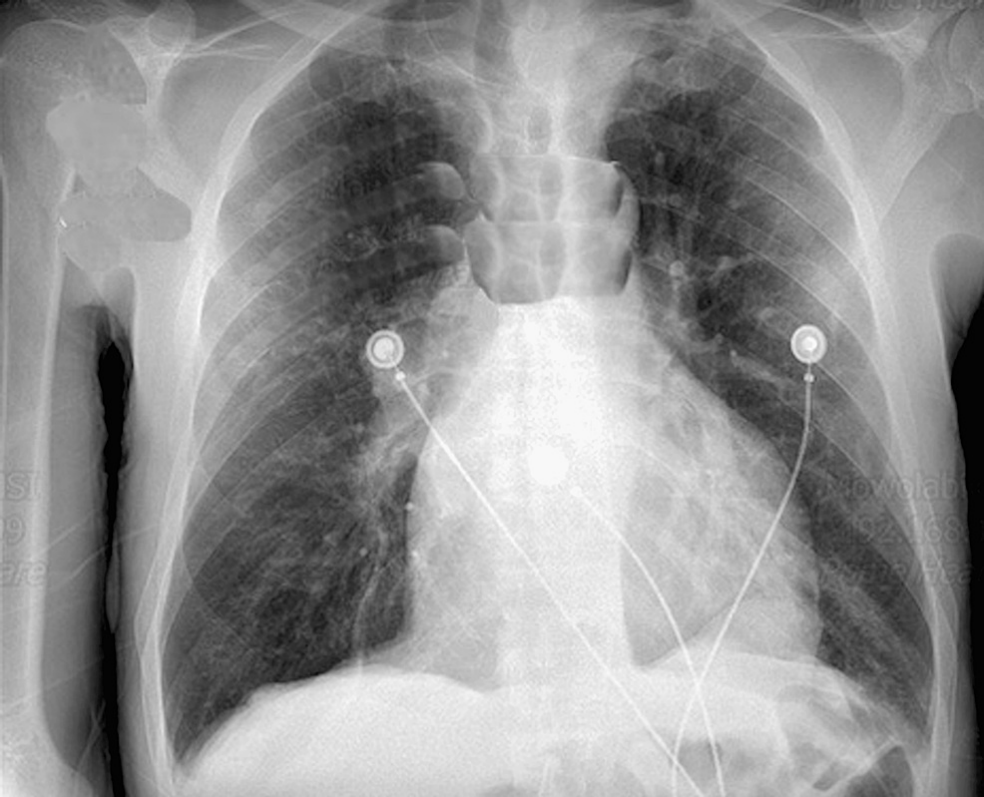
Chest X-ray showing no acute pathology.

**Figure 2:**
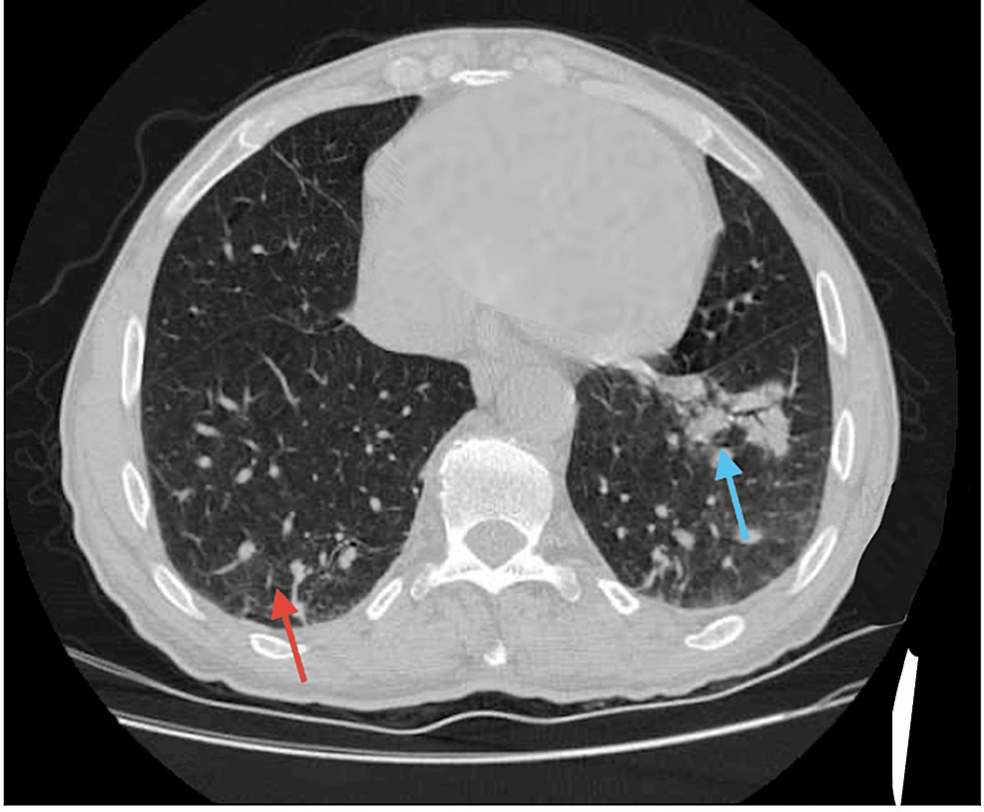
Computed tomography findings. The red arrow shows ground-glass opacities, and the blue arrow shows air bronchogram.

An 85-year-old male with a medical history of hypertension, chronic venous insufficiency, leg ulcers, deep vein thrombosis (DVT), aortic insufficiency, and hyperlipidemia presented to the Emergency Department with complaints of fatigue, lightheadedness, and dizziness for the past two days. The patient stated that the dizziness was aggravated by walking and relieved by sitting. The patient also stated that he was experiencing decreased appetite and decreased thirst for two days due to a strange taste in his mouth. He had received two doses of the BNT162b2 vaccine, the first dose on February 26, 2021, and the second dose on March 2, 2021. However, he did not receive his influenza vaccination. The patient denied fever, chills, shortness of breath (SOB), nausea, and vomiting. On admission, his body temperature was 97°F, blood pressure was 194/77 mmHg, heart rate (HR) was 92 beats/minute, respiratory rate (RR) was 16 breaths/minute, and he was saturating 100% on room air (RA). Physical examination was unremarkable. Chest X-ray (CXR) was also unremarkable (Figure 1). Electrocardiogram (ECG) showed normal sinus rhythm with left anterior fascicular block left ventricular hypertrophy (LVH). The computed tomography (CT) scan of the head without contrast was unremarkable. CT scan of the chest without contrast showed areas of infiltrates and consolidation within the left lower lobe. No pleural effusion or lymphadenopathy was noted (Figure 2).

Severe acute respiratory syndrome coronavirus 2 (SARS-CoV-2) reverse transcription-polymerase chain reaction (RT-PCR) and rapid influenza B antigen were positive. *Streptococcus pneumoniae* and *Legionella* antigen were negative. Sputum culture was not performed. All inflammatory markers, including ferritin, procalcitonin, C-reactive protein (CRP), lactate dehydrogenase (LDH), and D-dimer, were elevated, as shown in Table 1. Clinically, elevated procalcitonin and CT of the chest led to the diagnosis of bacterial pneumonia. The patient was given intravenous (IV) remdesivir for three days as he had a mild COVID-19 infection and we wanted to prevent the progression to severe disease according to the Infectious Diseases Society of America guidelines. Remdesivir 200 mg was given on day one and 100 mg for two days. oseltamivir 30 mg twice daily for five days, and oral levofloxacin 750 mg for day one, followed by 500 mg every 48 hours for seven days was also used in his management. The patient was discharged with outpatient follow-up.

**Table 1:** Initial laboratory data of the patient.

Laboratory parameters	Values	Reference range
Sodium	140	136–145 mmol/L
Potassium	4.2	3.5–5.3 mmol/L
Chloride	106	98–110 mmol/L
Blood urea nitrogen (BUN)	19	6–24 mg/dL
Creatinine	1.3	0.6–1.2 mg/dL
Aspartate transaminase	24	10–36 U/L
Alanine transaminase	11	9–46 U/L
White blood cell	3.10	4.4–11 × 10^3^/µL
Neutrophils (absolute)	2.1	1.7–7.0 × 10^3^/µL
Lymphocytes (absolute)	0.8	0.9–2.9 × 10^3^/µL
Monocytes (absolute)	0.2	0.3–0.9 × 10^3^/µL
Hemoglobin	10.4	13.5–17.5 g/dL
Platelets	138	150–450 × 10^3^/µL
D-dimer	1,273	0.0–500 ng/ml
High-sensitivity troponin I	54	0–76 ng/L
Procalcitonin	9.62	0–0.50 ng/ml
Lactate dehydrogenase	459	122–222 U/L
C-reactive protein	6.5	0.0–0.8 mg/dl
Ferritin	294	24–336 ng/ml

## Discussion

In medicine, co-infections often occur, such as hepatitis B and D co-infection and human immunodeficiency virus and hepatitis C co-infection. Co-infecting organisms might be of the same general type such as viruses or different such as a bacterium and a fungus or a virus and a bacteria like the case reported here [3]. The co-infection of COVID-19, influenza, and bacterial pneumonia, like any other co-infection, increases a patient’s probability of more complications such as sepsis and respiratory failure. While rare, the potential consequences of COVID-19, influenza, and bacterial pneumonia co-infection are ones that healthcare providers should be anticipating as the seasons change from summer to winter. COVID-19 and other respiratory pathogens, including bacteria, co-circulate in the environment. In this case report, we illustrated the clinical spectrum and disease severity of COVID-19, influenza, and bacterial pneumonia. The disease burden during this season due to laboratory-confirmed influenza as of November 10, 2022, according to the Centers for Disease Control and Prevention, is estimated to be 2.8 million illnesses, 23,000 hospitalizations, and 1,300 deaths from influenza, which is higher than that of the previous season [4]. This can be attributed to the reduction in preventive measures that were taken in the previous seasons such as wearing facial masks, social distancing, hand hygiene, and over-testing. The clinical presentation of the three pathogens can be similar. Patients with COVID-19 can present with a wide range of symptoms varying from mild symptoms, including, but not limited to, fever, chills, cough, SOB, fatigue, muscle or body aches, headache, loss of taste or smell, sore throat, congestion, runny nose, nausea, vomiting, and diarrhea. They can also present with severe symptoms such as multiorgan failure, acute respiratory distress syndrome, and renal failure. Symptoms begin to present two to 14 days after exposure to the virus [5]. Patients with the influenza virus might present similarly to those with COVID-19 with symptoms of fever, cough, sore throat, body aches, and fatigue. The main difference between patients with COVID-19 and influenza is the incubation period. Patients with influenza begin to experience symptoms one to four days after infection [6]. Patients with bacterial pneumonia can present similarly to patients who have COVID-19 and influenza with differences such as a confused mental state or delirium, productive cough, tachycardia, and stabbing chest pain that worsens with deep breathing or coughing [7]. The diagnostic modalities for the three diseases are similar, with history and physical examination guiding the laboratory and radiologic modalities. Initially, a viral test either a nucleic acid amplification test, most commonly an RT-PCR assay, or an antigen test can be used in the diagnosis of COVID-19 [8]. There are several tests offered to diagnose influenza viruses in respiratory specimens. The most common is rapid influenza diagnostic tests which provide results in 10-15 minutes but is not as sensitive as other flu tests. Therefore, if a patient tests negative, we can perform another test such as the rapid molecular assay, RT-PCR, viral culture, and immunofluorescence assays [9]. Patients with pneumonia can be diagnosed with chest X-rays, blood cultures, sputum cultures, chest CT scans, and pleural fluid cultures [7]. Procalcitonin has been shown to have great significance in distinguishing between bacterial and viral infections, and the turnaround time for results is within a couple of hours. The procalcitonin result has greater than 65% accuracy in distinguishing between patients who have viral from bacterial etiology in patients with community-acquired pneumonia [10].

## Conclusions

Patients with COVID-19, influenza, and bacterial pneumonia present similarly. It is essential that providers recognize coinfections early to manage patients appropriately and decrease morbidity and mortality. Patients with a co-infection of COVID-19, influenza, and bacterial pneumonia can be treated with remdesivir for COVID-19, oseltamivir for influenza, and appropriate antibiotics for bacterial pneumonia. Special attention should be paid to elderly patients and those with underlying health conditions to decrease severe consequences such as sepsis and respiratory failure.
